# JMJD6 Regulates ERα Methylation on Arginine

**DOI:** 10.1371/journal.pone.0087982

**Published:** 2014-02-03

**Authors:** Coralie Poulard, Juliette Rambaud, Nader Hussein, Laura Corbo, Muriel Le Romancer

**Affiliations:** 1 Université de Lyon, F-69000 Lyon, France; 2 Université Lyon 1, F-69000 Lyon, France; 3 Inserm U1052, Centre de Recherche en Cancérologie de Lyon, F-69000 Lyon, France; 4 CNRS UMR5286, Centre de Recherche en Cancérologie de Lyon, F-69000 Lyon, France; 5 Equipe Labellisée « La Ligue »; 6 Lebanese University, Faculty of Sciences, Doctoral School of Sciences and Technology, PRASE, Hadath, Lebanon; Institut de Génomique Fonctionnelle de Lyon, France

## Abstract

ERα functions are tightly controlled by numerous post-translational modifications including arginine methylation, which is required to mediate the extranuclear functions of the receptor. We report that upon oestrogenic stimulation, JMJD6, the only arginine demethylase described so far, interacts with and regulates methylated ERα (metERα) function. Moreover, by combining the silencing of JMJD6 with demethylation assays, we show that metERα is a new substrate for JMJD6. We propose that the demethylase activity of JMJD6 is a decisive regulator of the rapid physiological responses to oestrogen.

## Introduction

Oestrogen (17β-oestradiol, E_2_) a member of the steroid hormone family, plays a crucial role in many physiological processes and in disease, namely in breast cancer. The biological actions of oestrogen are mediated through ERα and ERβ, which function in the nucleus as ligand-dependent transcription factors promoting gene transcription and the stimulation of cell growth in various tissues, including breast epithelial cells [1], [2].

In addition to these well-documented effects, oestrogens also activate multiple signal transduction cascades outside of the nucleus via nongenomic signalling. This nongenomic pathway involves growth factor-dependent kinases and adaptor proteins leading to downstream activation of signalling molecules, such as MAPK and Akt [3]–[6]. Cellular responses to oestrogens are highly controlled and require the regulation of ERα function through numerous post-translational modifications that regulate both genomic and nongenomic pathways (For a review, [7]). Most nongenomic effects of oestrogen are mediated through the recruitment of the tyrosine kinase Src and PI3K [3], [4]. Our team has contributed to the understanding of this pathway by demonstrating that arginine methylation of the receptor is prerequisite to oestrogen-induced formation of the ERα/Src/PI3K complex which activates Akt [8]. Recently, we also showed that this pathway is activated in aggressive breast tumours and could constitute a new potential target for therapy [9].

Our finding of ERα methylation being a dynamic process strongly suggests the involvement of an enzyme that reverses this methylation. We therefore investigated if the only arginine demethylase identified so far, JMJD6, plays a role in this process. The enzymatic activity of this protein was described by Buick’s team on histones displaying asymmetrical as well as symmetrical demethylation on arginine residues [10]. Indeed, three types of arginine methylation exist in mammalian cells: monomethylarginine (MMA), asymmetric dimethylarginine (ADMA) and symmetric dimethylarginine (SDMA). PRMT1 is the major Type 1 arginine methyltransferase that adds the ADMA mark and PRMT5 is the principal Type 2 enzyme which catalyzes SDMA [11].

Recent publications have shown that JMJD6 also possesses lysyl hydroxydase activity [12]–[14].

In this paper, we demonstrate that JMJD6 demethylates the ERα methylated on R260, thereby regulating oestrogen nongenomic signalling. Moreover, global approaches suggest that JMJD6 regulates other arginine methylated-proteins and further studies should be done to validate this point, based on the data obtained by mass spectrometry.

## Results and Discussion

### JMJD6 Specifically Interacts with the Methylated Form of ERα

**Figure 1:**
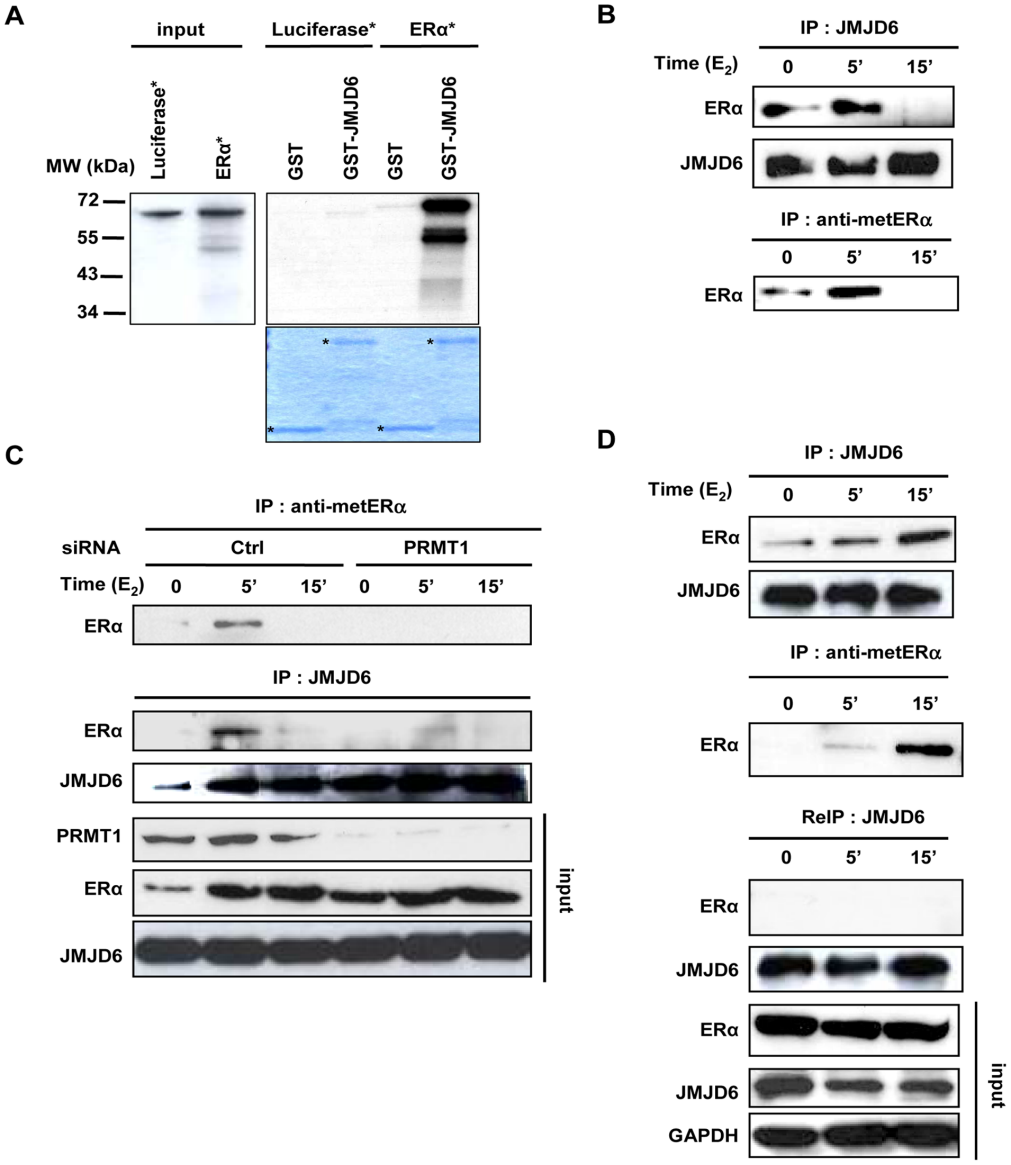
JMJD6 specifically interacts with the methylated form of ERα. (**A**) *In vitro* synthesized and radiolabelled ERα protein was incubated with GST-JMJD6 and binding was analysed by autoradiography. Luciferase was used as a negative control. The lower panel shows the corresponding coomassie staining. (**B**) MCF-7 were treated with E_2_ for the indicated times and the interaction of JMJD6 with ERα was analysed by immunoprecipitation. On the same extract methylated ERα (metERα) was studied by immunoprecipitation with the anti-metERα revealed with an anti-ERα. (**C**) PRMT1 is essential to JMJD6/ERα interaction. Lysates of MCF-7 cells transfected with control siRNAs or with specific PRMT1 siRNAs were treated with vehicle or E_2_ (10^−8^ M), analysed for ERα methylation and immunoprecipitated with the anti-JMJD6 antibody before analysis with the indicated antibodies. The amount of ERα, JMJD6 and PRMT1 in the different samples was determined by western blot in the input. (**D**) JMJD6 interacts specifically with metERα. MCF-7 treated with E_2_ as above were lysed and analysed for JMJD6/ERα interaction. In parallel, cell extracts were immunoprecipitated with metERα antibody. Unbound fractions were then used for a second immunoprecipitation using anti-JMJD6 antibody followed by western blotting with anti-ERα and anti-JMJD6 antibodies. The amount of ERα and JMJD6 in the different samples was determined by western blot in the input.

To investigate a functional link between JMJD6 and ERα, we firstly analysed whether the two proteins could interact. GST pull-down assays indicated that JMJD6 directly associates with ERα (Figure 1A) specifically within the hinge domain (Figure S1 A and B). To assess if JMJD6 interacts *in vitro* with metERα, we performed competition experiments adding the peptide containing R260 methylated or not within the reaction, however, it does not modify the interaction (Figure S2). Next, we wished to validate the binding between endogenous JMJD6 and ERα. Because ERα is rapidly methylated after E_2_ treatment, we performed reciprocal immunoprecipitation on MCF-7 cells treated with E_2_ for the indicated times (Figure 1B and Figure S3). Oestrogen treatment induced a rapid and transient interaction between ERα and JMJD6. Interestingly, the kinetics of interaction were reminiscent of those previously described for ERα methylation on R260 [8]. However, while the ERα methylation can vary between five and fifteen minutes after estrogen treatment along the experiments, the kinetics of interaction of ERα with JMJD6 were concomitant with the kinetic of ERα methylation, suggesting that JMJD6 could specifically interact with methylated ERα (metERα). Several additional findings supported this hypothesis: i) siRNA-mediated reduction of the arginine methyltransferase PRMT1 responsible for ERα methylation, impaired both ERα methylation and the ERα/JMJD6 interaction (Figure 1C); ii) depletion of metERα by immunoprecipitation using an antibody specifically recognizing the ERα methylated on R260, also disrupted the ERα/JMJD6 interaction (Figure 1D); and finally, iii) analysis in other breast cancer cell lines showed that the interaction of ERα with JMJD6 was restricted to cells expressing metERα, reinforcing the idea that JMJD6 interacts specifically with metERα (Figure S4).

Altogether these data clearly establish that contrarily to *in vitro* results showing the interaction between JMJD6 and ERα was estrogen independent, in cell lines JMJD6 interacts specifically with metERα upon E_2_ stimulation.

### JMJD6 Interacts with metERα within the Complex metERα/Src/PI3K

The methylation of ERα is a critical upstream signal, required to mediate the interaction of the receptor with Src and the p85 subunit of PI3K, which propagates the signal to downstream transduction cascades responsible for orchestrating cell proliferation and survival. Disappearance of the methylated ERα occurs concomitantly with the dissociation of the complex, leading to the extinction of downstream kinase activation [8].

**Figure 2:**
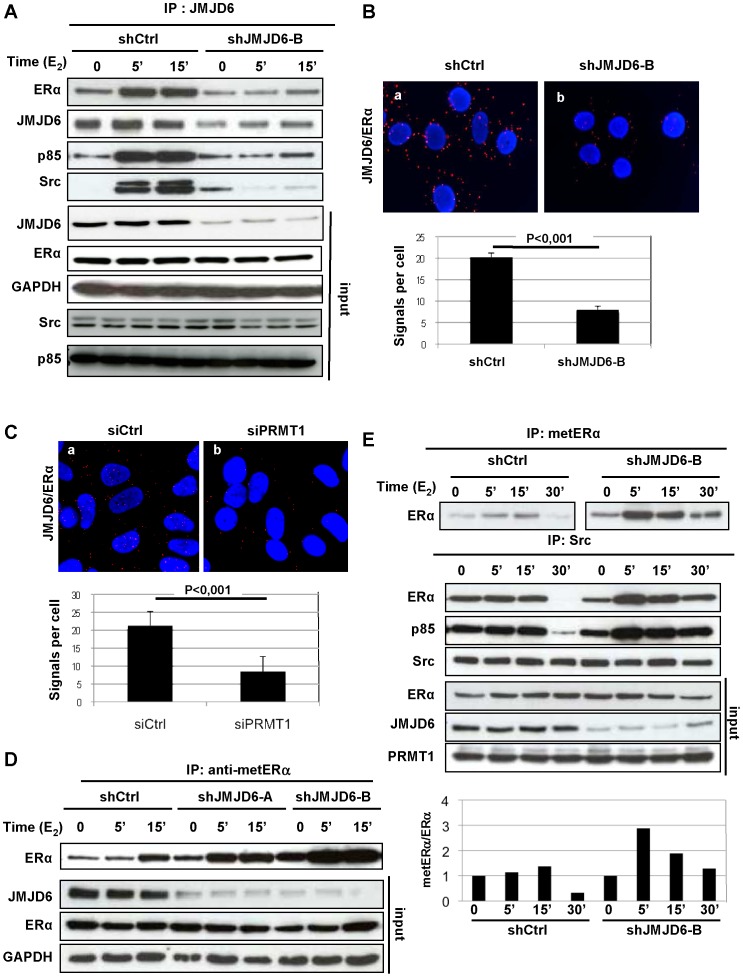
JMJD6 interacts with metERα within the complex metERα/Src/PI3K. (**A**) ERα, Src and PI3K coimmunoprecipitate with JMJD6. Lysates of MCF-7 cells, control cells (shCtrl), and JMJD6 knockdown cells (shJMJD6) were immunoprecipitated with JMJD6 antibody. The immunoprecipitates were then blotted with antibodies against PI3K (p85), Src, ERα and JMJD6. The amount of ERα, Src, p85 (PI3K) and GAPDH in the different samples was determined by western blot. (**B**) Detection of endogenous interaction between JMJD6 and ERα by PLA. MCF-7 cells were incubated with E_2_ 10^−8^ M for 5 min. After fixation, *in situ* PLA for JMJD6/ERα was performed using antibodies raised against JMJD6 and ERα (panels a,b). The detected dimers are represented by red dots. The nuclei were counterstained with DAPI (blue) (Obj: X63). The lower panel shows quantification of the number of signals per cell was performed by computer-assisted analysis as reported in the Materials and Methods. The mean +/− s.e.m. of four experiments is shown. P-value was determined by Student’s t-test. (**C**) JMJD6/ERα was analyzed by PLA as in B, in MCF-7 cells knocked down for PRMT1 (panel b) or control cells (panel a). The lower panel shows quantification of the number of signals per cell (**D**) ERα methylation was analysed after E_2_ stimulation on control (shCtrl) and shJMJD6 cells (as described in Figure 1B). The amount of ERα, JMJD6 and GAPDH in the different samples was determined by western blot. (**E**) ERα methylation was analysed after E_2_ stimulation on control and ShJMJD6 cells as in D and from the same extracts, immunoprecipitation with anti-src antibody was performed. The presence of ERα and p85 was then analysed. The amount of ERα, p85 and PRMT1 were determined in the inputs. The lower panel shows the relative quantification of metERα/ERα.

In view of these earlier findings, we wondered whether JMJD6 might participate towards regulating the E_2_ nongenomic signalling. To test this hypothesis, we performed co-immunoprecipitation experiments on E_2_-treated cells. We found that JMJD6 co-precipitated ERα with Src and PI3K. In addition, these interactions were clearly reduced in cells in which JMJD6 had been invalidated (shJMJD6) (Figure 2A). Interestingly, the kinetics of interactions were concomitant with the kinetics of ERα methylation suggesting that the JMJD6/metERα interaction occurs when the receptor is in complex with Src and PI3K (Figure 2A). We then examined in which compartment this interaction occurred by Proximity Ligation Assay (PLA). Figure 2B shows that JMJD6 interacted with ERα (panel a) in the cytoplasm of MCF-7 cells, as indicated by the presence of red dots. The signals strongly decreased in MCF-7 cells in which expression of JMJD6 was downregulated (Figure 2B, panel b). The interactions were quantified by counting the number of dots per cell (Figure 2B, lower panel). Moreover, knockdown of PRMT1 strongly decreased JMJD6/ERα interaction analyzed by PLA (Figure 2C), supporting that JMJD6 specifically interacts with methylated form of ERα which is localized in the cytoplasm of the cells [8]. The recruitment of JMJD6 seemed to be mainly mediated by ERα since JMJD6 could only weakly interact or not at all with Src or p85 of PI3K (Figure S5A and B). However, we cannot exclude that other cofactors are important to recruit JMJD6 within the complex.

To assess whether JMJD6 affects the methylation of ERα on R260, we used JMJD6-knocked down cells (shJMJD6). After E_2_ treatment, ERα methylation was strongly increased in shJMJD6 cells compared with in control cells (Figure 2D). However, ERα methylation still decreased after 30 min of E_2_ treatment probably due to the residual presence of JMJD6. Of note, this phenomenon occurred concomitantly with an increase in metERα/Src/PI3K complex (Figure 2E).

Taken together, these data strongly suggest that JMJD6, by regulating the methylation of ERα, could play a role in the oestrogen rapid signalling pathway.

### JMJD6 Preferentially Demethylates Asymmetric Dimethylation on Arginine Residues

**Figure 3:**
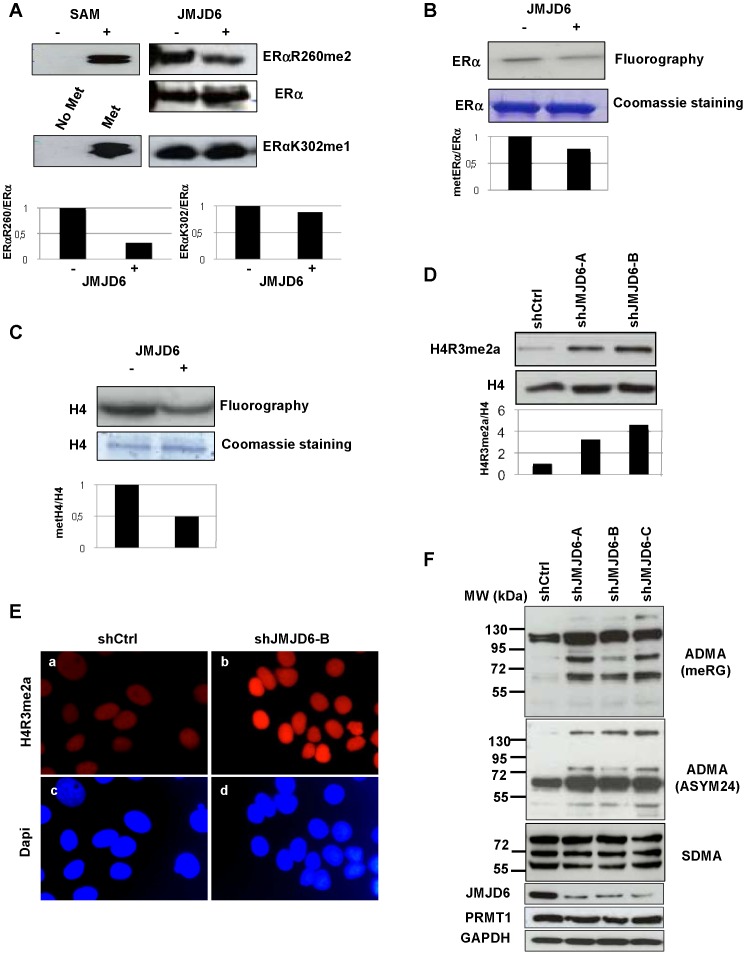
JMJD6 demethylates asymmetric dimethylation on arginine residues. (**A**) GST-ERα fragment (251–305) was methylated by PRMT1 in the presence or in absence of cold methyl donor, SAM. Then, a demethylation assay was performed adding GST (−) or GST-JMJD6 (+). Western blot was performed with the anti-ERαR260me2 antibody and an anti-ERα antibody recognizing the hinge domain (upper panel). Under the same conditions, demethylation was assessed on an ERα peptide methylated on K302 and recognized by the antibody anti- ERα K302me1. The lower panel shows the relative quantification of ERαR260me2/ERα and of ERαK302me1/ERα. (**B**) Hinge ERα was incubated with GST-PRMT1 in the presence of [methyl-^3^H] SAM. A demethylation assay was then performed in the presence of GST-JMJD6 or GST. Reaction products were analysed by SDS-PAGE followed by fluorography. The coomassie staining of hinge ERα is also shown. The lower panel shows the relative quantification of metERα/ERα. (**C**) The experiment of demethylation was performed for H4 methylation as in B (**D**) Acid extracted-histones from MCF-7 cells, control cells (shCtrl) and JMJD6 knockdown cells (shJMJD6A and B) were analysed by western blot with an antibody recognizing specifically the asymmetric methylation of arginine 3 of Histone H4 anti-H4R3me2a. Control western blotting was performed with anti-histone H4. The lower panel shows the relative quantification of H4R3me2a/H4. (**E**) H4R3 dimethylation was also analysed by immunofluorescence in the same cells as described above (panel a,b). DAPI staining is shown in panels c and d. (**F**) Lysates of control and shJMJD6 cells were analysed by western blotting with antibodies recognizing asymmetric dimethylation (MeRG and ASYM24) and symmetric dimethylation (SYM10). Controls were performed with anti PRMT1, JMJD6 and GAPDH antibodies.

In light of our finding that JMJD6 binds to metERα and regulates its methylation, we wondered whether JMJD6 could demethylate ERα *in vitro*. We performed a demethylation assay using recombinant JMJD6 on ERα methylated *in vitro* by PRMT1 as already described [8]. Using a specific antibody directed against ERα asymmetrically methylated on R260 (Figure 3A left upper panel), we found that JMJD6 reduced the methylation of ERα R260 (Figure 3A, right panel). We obtained the same demethylation results when we used [^3^H]. AdoMet for the methylation assay and the methylation was visualized by autoradiography excluding possible artefactual antibody detection (Figure 3B).

However, as described by Bruick, its enzymatic activity remained faint, probably due to the need of a coactivator to potentiate its activity, as has been described for PRMT5 [15], [16] and for the lysine demethylase LSD1 [17]. In the same type of assay, JMJD6 was not able to demethylate the peptide of ERα containing monomethylated K302 (K302me1) [18] (Figure 3A, lower panel), thus highlighting the specificity of the catalytic activity of the recombinant protein.

Considering that histone H4 at arginine 3 (H4R3) is a substrate of JMJD6 [10] and that JMJD6 is able to demethylate ERα *in vitro* as shown above, we tested whether JMJD6 can remove dimethylation from histone H4 methylated *in vitro* by recombinant PRMT1. As shown in Figure 3C, JMJD6 was indeed able to decrease H4R3 ADMA *in vitro*. These results support the role of JMJD6 in the demethylation of the ADMA R3 residue on histone H4.

After validation of H4R3me2a antibody (Figure S6), concordant results were obtained in our *in vivo* test in which, strikingly, knockdown of JMJD6 significantly increased H4R3me2a, detected by a specific antibody, when compared with the control cells (Figure. 3D, compare lane 2 and 3 to lane 1), while the histone H4 level remained unchanged. Again, immunofluorescence analysis showed an enhanced staining of H4R3me2a in the nuclei of knocked down cells compared to control (Figure 3E).

To provide further evidence for the demethylating function of JMJD6, we used two complementary approaches. Firstly, we analysed the global ADMA of total proteins in MCF-7 cells depleted for JMJD6. Cell lysates of cells invalidated for JMJD6 with three different shRNAs were immunoblotted with an antibody recognizing specifically ADMA (meRG). As shown in Figure 3F, knockdown of JMJD6 had a strong effect on the amount of dimethylated proteins (compare lanes 2–4 to lane 1). We confirmed these results with another antibody recognizing ADMA (ASYM24). However, the same cell extracts analyzed with an antibody directed against SDMA did not show any difference in the amount of methylated proteins, suggesting that JMJD6 may have more affinity for target asymmetric dimethylation (Figure 3E). Of note, the overexpression of JMJD6 reduced global asymmetrical dimethylation assessed with ASYM24 antibody (Figure S7).

Next, we identified putative JMJD6 partners by immunoprecipitation of MCF-7 transfected with JMJD6, coupled to mass spectrometry analysis. In agreement with data in the literature [14], [19], [20] most of the identified proteins were RNA binding proteins implicated in RNA processing. Interestingly, 23/25 proteins identified have been shown to be methylated on arginine residues (Table 1). Of interest, one of the identified putative partners of JMJD6 was the arginine methyltransferase CARM1, adding further evidence for its role in ADMA regulation. This interaction has been validated by immunoprecipitation (Figure S8).

**Table 1:** Potential interacting proteins of JMJD6 identified by affinity purification and mass spectrometry.

**hnRNP U-like protein 1**	**Q9BUJ2**	**mRNA processing**	**DMA, MMA**	[24]
**hnRNP K**	**P61978**	**mRNA processing**	**DMA, MMA**	[24], [25]
**hnRNP L**	**P14866**	**mRNA processing**	**MMA**	[26]
**hnRNP Q**	**O60506**	**mRNA processing**	**MMA, DMA**	[26]
**U1 snRNP 70**	**P08621**	**mRNA processing**	**MMA**	[26]
**RNA helicase (DDX3)**	**O00571**	**mRNA processing**	**DMA, MMA**	[26]
**RNA helicase (DDX5)**	**P17844**	**mRNA processing**	**DMA, MMA**	[24]
**RNA helicase (DDX17)** *	**Q92841**	**mRNA processing**	**MMA**	[26]
**Paraspeckle protein 2 (PSP2)**	**Q96PK6**	**mRNA processing**	**MMA**	[26]
**PTB-associated-splicing factor (SFPQ)**	**P23246**	**mRNA processing**	**DMA, MMA**	[24], [27]
**Non-POU domain-containing octamer-binding protein**	**Q15233**	**mRNA processing**	**MMA**	[26]
**RNA-binding protein (FUS)**	**P35637**	**mRNA processing**	**DMA, MMA**	[24], [27]
**RNA-binding protein (EWS)**	**Q01844**	**mRNA processing**	**DMA, MMA**	[28], [29]
**Splicing factor HCC1** *	**Q14498**	**mRNA processing**	**DMA, MMA**	[24]
Poly(U)-binding-splicing factor(PUF60)*	Q9UHX1	mRNA processing		
**Paraspeckle component 1 (PSPC1)**	**Q8WXF1**	**transcription**	**DMA, MMA**	[24]
**TATA-BP-associated factor 2N TAF15**	**Q92804**	**transcription**	**DMA, MMA**	[24], [27]
**Arg-methyltransferase (CARM1)**	**Q86X55**	**transcription**	**DMA, MMA**	[30]
**Poly(A)-binding protein 1 (PABP1)**	**P11940**	**translation**	**DMA, MMA**	[24]
**Poly(A)-binding protein 4 (PABP4)**	**Q13310**	**Translation**	**DMA**	[27]
**Ras GTPase-activating BP1**	**Q13283**	**translation**	**DMA, MMA**	[24]
**SMARCD2**	**Q92925**	**Chromatin structure**	**DMA**	[26]
**nucleolin**	**P19338**	**mRNA processing/nucleolus**	**DMA**	[31]
Protein transport protein Sec23B	Q15437	Endosome trafficking		
**TFG**	**Q92734**	**unknown**	**DMA, MMA**	[24]

MMA: monomethylarginine. DMA: dimethylarginine. The bold characters highlight arginine methylated proteins.

*indicate proteins already identified as JMJD6 putative partners [14].

Altogether, these data support the notion that JMJD6 has a dual enzymatic role, as arginine demethylase and hydroxylase, and participate in various regulation pathways. Significantly, our results have shown that JMJD6 may function as an arginine demethylase for histone and, for the first time, for a non-histone target. Specifically JMJD6 negatively regulates ERα nongenomic signalling. After E_2_ treatment the methylation of ERα at R260 is crucial to propagate the signal to downstream transduction cascades. We therefore propose that the demethylase activity of JMJD6 is a decisive regulator of the rapid physiological responses to oestrogen, inducing the extinction of downstream kinases activation. Knowing that JMJD6 has been demonstrated to be a factor of poor prognosis in breast cancer [21], we could hypothesize that its demethylase activity could be involved in this effect maybe in part by regulating metERα/Src/PI3K/Akt signalling.

## Materials and Methods

### Plasmids and GST-fusion Proteins

GST-parallel-JMJD6 was a gift from Bruick [10]. GST-PRMT1, GST-ER176-302, GST-ER297-595, GST-ER176-251, GST-ER251-305 and GST-ER251-305 were produced as described [22].

### Cell Culture and Transfections

MCF-7, ZR75-1 and Cama-1 mammary cells were maintained at 37°C in the appropriate medium supplemented with 10% foetal calf serum 1% non-essential amino acids. Prior to experiments, cells were grown for 48 hr in phenol red-free medium and 10% charcoal-treated serum (Biowest). MCF-7, ZR75-1 and Cama-1 cells come from ATCC. MCF-7 and ZR75-1 have been certified by CelluloNet, Lyon, France.

The siRNA sequences targeting PRMT1 correspond to the coding regions 650–668 and have already been described [8]. 50 nM of PRMT1 specific siRNAs or the scrambled siRNA (Eurogentec) were transfected into MCF-7 cells (1×10^6^) using lipofectamine 2000 reagent (Invitrogen) according to the manufacturer’s guidelines. 72 hr after transfection, cells were lysed in RIPA buffer containing 50 mM Tris HCl, pH 8, 150 mM NaCl, 1 mM EDTA, 1% NP-40, and 0.25% deoxycholate.

### Antibodies

See Table 2.

**Table 2:** List of antibodies and their application.

Antibodies	Species	Company	DilutionWB	Dilution IF/PLA
GAPDH (H86504M)	Mouse	BD International	1/25000	
ERα (04-820)	Rabbit	Millipore	1/1000	
ER (sc-8005)	Mouse	Santa Cruz	1/1000	
ERα (sc-544)	Rabbit	Santa Cruz	1/500	
JMJD6 (ab10526)	Rabbit	ab10526 Abcam	1/1000	1/400
JMJD6 (sc-28348)	Mouse	Santa Cruz	1/500	
PRMT1, (24333)	Rabbit	Millipore	1/4000	
H4R3me2as (#39705)	Rabbit	Active motif	1/1000	1/200
meRG (#800002)	Rabbit	CH_3_ Biosystems	1/1000	
Asym24 (07-414)	Rabbit	Millipore	1/4000	
Sym10 (07-412)	Rabbit	Millipore	1/1000	
ERαR260me2 or metERα	Mouse	Home made	1/1000	
PI3K p85 (06-195)	Rabbit	Millipore	1/9000	
ERαK302me1	Rabbit	Gift from P. Vertino	1/5000	
Src (sc-8056)	Mouse	Santa Cruz	1/1000	1/150
Src (2109)	Rabbit	Cell Signaling Technology	1/1000	

### JMJD6 Stable Knockdown Using Lentiviral Short Hairpin RNA

Three pre-made lentiviral short hairpin RNA (shRNA) constructs targeting human JMJD6 and one negative control construct created in the same vector backbone (pLKO.1-Puro) were purchased from SIGMA. Puromycin selection (1 µg/mL) was started 48 h after lentiviral infection.

### GST-pull-down Assay

Psg5-ERα and pCDNA3-JMJD6 plasmid were transcribed and translated *in vitro* using T7-coupled reticulocyte lysate (Promega), in the presence of [^35^S]methionine. Labelled proteins were incubated with 10 µg of purified recombinant GST- fusion proteins in 200 µl of binding buffer (Tris 20 mM pH 7.4, NaCl 0.1 M, EDTA 1 mM, glycerol 10%, Igepal 0.25%) with 1 mM DTT and 1% milk) for 2 h at room temperature. In competition experiments, peptide containing R260 of 1 µM of ERα methylated or not was added to the reactions [8]. Beads were washed three times in binding buffer, and then bound proteins were resolved on SDS-polyacrylamide gel electrophoresis (PAGE), and visualized by autoradiography.

### Affinity Purification and Mass Spectrometry

MCF-7 cells were transiently transfected with V5-tagged JMJD6. Cell extracts were purified with agarose beads coupled to V5 antibody and loaded on SDS-PAGE. After elution, proteins were digested by trypsin and peptides were analysed by LC/MS/MS at IBCP, Lyon.

### Immunoprecipitation and Western Blotting

To study the effect of oestrogen, the cells were treated for different times with E_2_ (Sigma). After treatment, cells were lysed in RIPA buffer. Protein extracts were incubated with primary antibodies overnight at 4°C with shaking. Protein A-agarose or protein L-agarose beads were added and incubated 1 h at 4°C. The immunoprecipitates were separated on SDS-PAGE. The proteins were visualized by an enhanced chemiluminescence kit (Roche Molecular Biochemicals) following the manufacturer’s instructions.

For histone extraction, cells were lysed in a solution containing 3% of SDS and 10% of β-mercaptoethanol for 2 min at 95°C, then cooled in ice. After addition of DNAase, the samples were loaded on SDS-PAGE.

Immunoblot images were digitized and quantified using the Image J Software.

### Methylation and Demethylation Assays

The *in vitro* methylation protocol has been described previously [8]. The demethylation assay was performed as described for methylated histones [10]. We also used GST-hinge-ERα or histone H4 methylated by PRMT1 and purified on microspin columns to remove S adenosylmethionine (SAM) or a peptide corresponding to amino acids 297–308 non methylated or monomethylated on K302 [18]. Some experiments were performed with [^3^H ]SAM and others with cold SAM. Then, GST-JMJD6 or GST was incubated at 37°C for 1 h and the reaction products were assayed by western blot analysis or by autoradiography.

### Proximity Ligation Assay

This technology developed by Olink Bioscience (Sweden) allows the visualisation of protein/protein interactions *in situ* and was first published in 2006 [23]. The experiments were performed following the manufacturer’s instructions and have been previously described [9].

### Image Acquisition and Analysis

The hybridized fluorescent slides were viewed under a Leica DM6000B microscope. Images were acquired under identical conditions at objective X63. For each sample, at least one hundred cells were counted. Analyses and quantifications of these samples were performed using Image J software.

## Supporting Information

Figure S1 Identification of domains of ERα interacting with JMJD6. A) The organization of ERα protein showing the functional domains ERα displays conserved functional domains. A/B including AF-1 (Activation Function 1), C containing the DBD (DNA binding domain), D called Hinge domain including nuclear localization signals, E containing the LBD (Ligand binding domain) and AF-2 (Activation Function 2) and F allowing agonist/antagonist regulation. B) Radioactive JMJD6 (*) was incubated with GST and with the different domains of ERα coupled with GST, and the bound proteins were visualized by autoradiography. The lower panel shows the coomassie staining of the gel.
(DOC)

Figure S2 Competition experiment of metERα peptide with ERα/GST-JMJD6 interaction. Radioactive ERα (*) was incubated with GST or GST-JMJD6 in the presence or in the absence of the peptide containing metERα (already described in [8].) and the bound proteins were visualized by autoradiography. The lower panel shows the coomassie staining of the gel. * indicates the different GST proteins.
(DOC)

Figure S3 JMJD6/ERα interaction in MCF-7cells. Immunoprecipitation was performed from E_2_-treated MCF-7 cell extracts with anti-ERα antibody and revealed with anti-ERα and anti-JMJD6 antibodies.
(DOC)

Figure S4 JMJD6/ERα interaction in human breast cancer cells. ZR75-1 (A), and Cama-1 (B) cells were analyzed for ERα methylation and JMJD6/ERα interaction. Immunoprecipitation of JMJD6 from extracts of estrogen-deprived cells (t = 0) stimulated with 10^−8^ M E_2_ for the indicated times was performed followed by western blotting with antibody against ERα and JMJD6. On the same extract metERα was analyzed by immunoprecipitation with the anti metERα revealed with an anti-ERα. PRMT1 expression was also analyzed by western blotting.
(DOC)

Figure S5 JMJD6/Src and JMJD6/PI3K interaction in vitro. A) GST pull down assay of *in vitro* translated ^35^S-labeled Src or p85 (PI3K) (*) was incubated with GST and GST-JMJD6 and the bound proteins were visualized by autoradiography. Luciferase was used as a negative control. B) The same experiments were performed in the presence or in absence of *in vitro* translated cold ERα to investigate if ERα could be the bridge mediating the interactions. The lower panel shows the coomassie staining of the gel. * indicates the different GST proteins.
(DOC)

Figure S6 Validation of anti-H4R3me2a specificity. Extracts from MCF-7 cells transfected with scrambled siRNA or siRNA targeting PRMT1 were assessed by western blotting for Histone H4 methylation using the anti-H4R3me2a. Controls were performed using anti-histone H4 and anti-PRMT1 antibodies.
(DOC)

Figure S7 Role of JMJD6 on global arginine methylation. MCF-7 cells were transfected with pcDNA3 empty vector or pCDNA3-JMJD6-V5. Cell extracts were analyzed by western blotting with an antibody recognizing asymmetric dimethylation (ASYM24). Controls were performed with anti-JMJD6, PRMT1 and GAPDH antibodies. A shorter exposition of the gel is shown in the right-hand panel (*). The lower panel shows quantification of protein methylation in cells transfected with JMJD6 versus mock.
(DOC)

Figure S8 Interaction between JMJD6 and CARM1. MCF-7 cells were transfected with pcDNA3 empty vector or pCDNA3-JMJD6-V5. Cell extracts were immunoprecipitated with V5 antibody and revealed for the presence of JMJD6 and CARM1 with the corresponding antibodies.
(DOC)
