# Supplementary material for: JMJD6 Regulates ERα Methylation on Arginine
Source: PLoS One. 2014 Feb 3;9(2):e87982. doi: 10.1371/journal.pone.0087982 (PMC3912157; doi:10.1371/journal.pone.0087982)
Supplement: Figure S1 — Identification of domains of ERα interacting with JMJD6. A) The organization of ERα protein showing the functional domains ERα displays conserved functional domains. A/B including AF-1 (Activation Function 1), C containing the DBD (DNA binding domain), D called Hinge domain including nuclear localization signals, E containing the LBD (Ligand binding domain) and AF-2 (Activation Function 2) and F allowing agonist/antagonist regulation. B) Radioactive JMJD6 (*) was incubated with GST and with the different domains of ERα coupled with GST, and the bound proteins were visualized by autoradiography. The lower panel shows the coomassie staining of the gel. (DOC) [file pone.0087982.s001.doc]

**Figure S1: Identification of domains of ERα interacting with JMJD6.**

A) The organization of ERα protein showing the functional domains ERα displays conserved functional domains. A/B including AF-1 (Activation Function 1), C containing the DBD (DNA binding domain), D called Hinge domain including nuclear localization signals, E containing the LBD (Ligand binding domain) and AF-2 (Activation Function 2) and F allowing agonist/antagonist regulation.

B) Radioactive JMJD6 (*) was incubated with GST and with the different domains of ERα coupled with GST, and the bound proteins were visualized by autoradiography.The lower panel shows the coomassie staining of the gel.
