# Supplementary material for: JMJD6 Regulates ERα Methylation on Arginine
Source: PLoS One. 2014 Feb 3;9(2):e87982. doi: 10.1371/journal.pone.0087982 (PMC3912157; doi:10.1371/journal.pone.0087982)
Supplement: Figure S2 — Competition experiment of metERα peptide with ERα/GST-JMJD6 interaction. Radioactive ERα (*) was incubated with GST or GST-JMJD6 in the presence or in the absence of the peptide containing metERα (already described in [8].) and the bound proteins were visualized by autoradiography. The lower panel shows the coomassie staining of the gel. * indicates the different GST proteins. (DOC) [file pone.0087982.s002.doc]

**Figure S2. Competition experiment of metERα peptide with ERα/GST-JMJD6 interaction.**

Radioactive ERα (*) was incubated with GST or GST-JMJD6 in the presence or in the absence of the peptide containing metERα (already described in [8].) and the bound proteins were visualized by autoradiography.The lower panel shows the coomassie staining of the gel. * indicates the different GST proteins.
