# Supplementary material for: JMJD6 Regulates ERα Methylation on Arginine
Source: PLoS One. 2014 Feb 3;9(2):e87982. doi: 10.1371/journal.pone.0087982 (PMC3912157; doi:10.1371/journal.pone.0087982)
Supplement: Figure S3 — JMJD6/ERα interaction in MCF-7cells. Immunoprecipitation was performed from E2-treated MCF-7 cell extracts with anti-ERα antibody and revealed with anti-ERα and anti-JMJD6 antibodies. (DOC) [file pone.0087982.s003.doc]

**Figure S3: JMJD6/ERα interaction in MCF-7cells.**

Immunoprecipitation was performed from E_2_-treated MCF-7 cell extracts with anti-ERα antibody and revealed with anti-ERα and anti-JMJD6 antibodies
