# Supplementary material for: JMJD6 Regulates ERα Methylation on Arginine
Source: PLoS One. 2014 Feb 3;9(2):e87982. doi: 10.1371/journal.pone.0087982 (PMC3912157; doi:10.1371/journal.pone.0087982)
Supplement: Figure S4 — JMJD6/ERα interaction in human breast cancer cells. ZR75-1 (A), and Cama-1 (B) cells were analyzed for ERα methylation and JMJD6/ERα interaction. Immunoprecipitation of JMJD6 from extracts of estrogen-deprived cells (t = 0) stimulated with 10−8 M E2 for the indicated times was performed followed by western blotting with antibody against ERα and JMJD6. On the same extract metERα was analyzed by immunoprecipitation with the anti metERα revealed with an anti-ERα. PRMT1 expression was also analyzed by western blotting. (DOC) [file pone.0087982.s004.doc]

**Figure S4: JMJD6/ERα interaction in human breast cancer cells.**

ZR75-1 (A), and Cama-1 (B) cells were analyzed for ERα methylation and JMJD6/ERα interaction. Immunoprecipitation of JMJD6 from extracts of estrogen-deprived cells (t=0) stimulated with 10^-8^M E_2_ for the indicated times was performed followed by western blotting with antibody against ERα and JMJD6. On the same extract metERα was analyzed by immunoprecipitation with the anti metERα revealed with an anti-ERα. PRMT1 expression was also analyzed by western blotting.
