# Supplementary material for: JMJD6 Regulates ERα Methylation on Arginine
Source: PLoS One. 2014 Feb 3;9(2):e87982. doi: 10.1371/journal.pone.0087982 (PMC3912157; doi:10.1371/journal.pone.0087982)
Supplement: Figure S5 — JMJD6/Src and JMJD6/PI3K interaction in vitro. A) GST pull down assay of in vitro translated 35S-labeled Src or p85 (PI3K) (*) was incubated with GST and GST-JMJD6 and the bound proteins were visualized by autoradiography. Luciferase was used as a negative control. B) The same experiments were performed in the presence or in absence of in vitro translated cold ERα to investigate if ERα could be the bridge mediating the interactions. The lower panel shows the coomassie staining of the gel. * indicates the different GST proteins. (DOC) [file pone.0087982.s005.doc]

**Figure S5: JMJD6/Src and JMJD6/PI3K interaction in vitro.**

A) GST pull down assay of *in vitro* translated ^35^S-labeled Src or p85 (PI3K) (*) was incubated with GST and GST-JMJD6 and the bound proteins were visualized by autoradiography. Luciferase was used as a negative control. B) The same experiments were performed in the presence or in absence of *in vitro* translated cold ERα to investigate if ERα could be the bridge mediating the interactions. The lower panel shows the coomassie staining of the gel. * indicates the different GST proteins.
