# Supplementary material for: JMJD6 Regulates ERα Methylation on Arginine
Source: PLoS One. 2014 Feb 3;9(2):e87982. doi: 10.1371/journal.pone.0087982 (PMC3912157; doi:10.1371/journal.pone.0087982)
Supplement: Figure S6 — Validation of anti-H4R3me2a specificity. Extracts from MCF-7 cells transfected with scrambled siRNA or siRNA targeting PRMT1 were assessed by western blotting for Histone H4 methylation using the anti-H4R3me2a. Controls were performed using anti-histone H4 and anti-PRMT1 antibodies. (DOC) [file pone.0087982.s006.doc]

**Figure S6: Validation of anti-H4R3me2a specificity.**

Extracts from MCF-7 cells transfected with scrambled siRNA or siRNA targeting PRMT1 were assessed by western blotting for Histone H4 methylation using the anti-H4R3me2a. Controls were performed using anti-histone H4 and anti-PRMT1 antibodies.
