# Supplementary material for: JMJD6 Regulates ERα Methylation on Arginine
Source: PLoS One. 2014 Feb 3;9(2):e87982. doi: 10.1371/journal.pone.0087982 (PMC3912157; doi:10.1371/journal.pone.0087982)
Supplement: Figure S7 — Role of JMJD6 on global arginine methylation. MCF-7 cells were transfected with pcDNA3 empty vector or pCDNA3-JMJD6-V5. Cell extracts were analyzed by western blotting with an antibody recognizing asymmetric dimethylation (ASYM24). Controls were performed with anti-JMJD6, PRMT1 and GAPDH antibodies. A shorter exposition of the gel is shown in the right-hand panel (*). The lower panel shows quantification of protein methylation in cells transfected with JMJD6 versus mock. (DOC) [file pone.0087982.s007.doc]

**Figure S7: Role of JMJD6 on global arginine methylation.**

MCF-7 cells were transfected with pcDNA3 empty vector or pCDNA3-JMJD6-V5. Cell extracts were analyzed by western blotting with an antibody recognizing asymmetric dimethylation (ASYM24). Controls were performed with anti-JMJD6, PRMT1 and GAPDH antibodies. A shorter exposition of the gel is shown in the right-hand panel (*). The lower panel shows quantification of protein methylation in cells transfected with JMJD6 versus mock.
