# Supplementary material for: JMJD6 Regulates ERα Methylation on Arginine
Source: PLoS One. 2014 Feb 3;9(2):e87982. doi: 10.1371/journal.pone.0087982 (PMC3912157; doi:10.1371/journal.pone.0087982)
Supplement: Figure S8 — Interaction between JMJD6 and CARM1. MCF-7 cells were transfected with pcDNA3 empty vector or pCDNA3-JMJD6-V5. Cell extracts were immunoprecipitated with V5 antibody and revealed for the presence of JMJD6 and CARM1 with the corresponding antibodies. (DOC) [file pone.0087982.s008.doc]

**Figure S8: Interaction between JMJD6 and CARM1.**

MCF-7 cells were transfected with pcDNA3 empty vector or pCDNA3-JMJD6-V5. Cell extracts were immunoprecipitated with V5 antibody and revealed for the presence of JMJD6 and CARM1 with the corresponding antibodies.
